# Effects of Larval Density on *Plutella xylostella* Resistance to Granulosis Virus

**DOI:** 10.3390/insects11120857

**Published:** 2020-12-02

**Authors:** Hailong Kong, Zhonglin Liu, Pingjun Yang, Lin Yuan, Wanghui Jing, Chuanlei Dong, Minyuan Zheng, Zhen Tian, Qiuli Hou, Shude Zhu

**Affiliations:** 1 College of Horticulture and Plant Protection, Yangzhou University, Wenhui East Road, NO. 48, Yangzhou 225009, China; MZ120201303@yzu.edu.cn (Z.L.); MZ120190999@yzu.edu.cn (L.Y.); MX120180617@yzu.edu.cn (W.J.); DX120200126@yzu.edu.cn (C.D.); MZ160724@yzu.edu.cn (M.Z.); 006783@yzu.edu.cn (Z.T.); houql@yzu.edu.cn (Q.H.); sdzhu@yzu.edu.cn (S.Z.); 2 Suzhou Plant Protection and Plant Quarantine Station, Stadium Road, NO. 4, Suzhou 215006, China; cbk@jsagri.gov.cn

**Keywords:** *Plutella xylostella*, larval density, granulosis virus, larval resistance, immune response

## Abstract

**Simple Summary:**

Generally, the transmission of pathogens is positively density-dependent; therefore, the risk of insects becoming infected by diseases increases with increasing population density. It has been reported that some phase-polyphenic insects from high-density conditions are more resistant (or susceptible or identical) to pathogens than those from low-density conditions. This phenomenon is termed “density-dependent prophylaxis” (DDP). The diamondback moth (DBM), *Plutella xylostella*, one of the most destructive insect pests affecting cruciferous crops, is non phase-polyphenic. Biological control, especially by pathogens, plays an important role in the integrated pest management program of DBM. However, whether the *P. xylostella* larval population exhibits DDP has not been elucidated. The resistance of DBM larvae to *P. xylostella* granulosis virus (*Plxy* GV) and their immune response to the virus under different density treatments were investigated under laboratory conditions. Our results demonstrated that *P. xylostella* larvae exhibited DDP within a certain limited density. This study may help to elucidate the biocontrol effect of different density populations of *P. xylostella* by granulosis virus and guide improvements in future management strategy.

**Abstract:**

It has been reported that some phase-polyphenic insects from high-density conditions are more resistant to pathogens than those from low-density conditions. This phenomenon is termed “density-dependent prophylaxis” (DDP). However, whether non phase-polyphenic insects exhibit DDP has rarely been elucidated. The diamondback moth (DBM), *Plutella xylostella*, one of the most destructive insect pests affecting cruciferous crops, is non phase-polyphenic. In this study, the resistance of DBM larvae to *P. xylostella* granulosis virus (*Plxy* GV) and their immune response to the virus when reared at densities of 1, 2, 5, 10, 15, and 20 larvae per Petri dish were investigated under laboratory conditions. Compared with larvae reared at lower densities, larvae reared at moderate density showed a significantly higher survival rate, but the survival rate significantly decreased with further increases in rearing density. Furthermore, the phenoloxidase, lysozyme and antibacterial activity and total hemocyte count in the hemolymph of the larvae, regardless of whether they were challenged with the virus, from different larval densities corresponded to the observed differences in resistance to *Plxy* GV. These results demonstrated that *P. xylostella* larvae exhibited DDP within a certain limited density. This study may help to elucidate the biocontrol effect of different density populations of *P. xylostella* by granulosis virus and guide improvements in future management strategy.

## 1. Introduction

Insects infected by pathogens are ubiquitous in nature [1]. Generally, the transmission of pathogens (or parasites) is positively density-dependent; therefore, the risk of insects becoming infected by diseases increases with increasing population density [2]. It has been found that some insects use early population density to predict the risk of pathogen attack and accordingly allocate resources to increase pathogen resistance [3]. That is, individuals from high-density conditions are predicted to be more resistant to pathogens (or parasites) than those from low-density conditions. This phenomenon is termed “density-dependent prophylaxis” (DDP), and it has been reported in some phase-polyphenic insect species, including *Spodoptera exempta* [4], *Spodoptera littoralis* [4], *Mythimna separata* [1], and *Locusta migratoria* [5]. Meanwhile, an enhanced immune response is also observed in these species under high-density conditions. The phenoloxidase (PO) enzyme system plays a key role in the insect immune response. PO activity was observed to be significantly increased with larval density in *S. littoralis*, *S. exempta*, and *M. separata* [1,4]. Increases in total hemocyte counts and antibacterial activity were also observed in *M. separata* [1].

However, some insects living in high-density populations might tailor their immune responses due to limited resources and physiological stress. Thus, a second hypothesis is that high density results in insects that are more susceptible to pathogens than less crowded insects. Piesk et al. (2013) [6] found that immune parameters, including phenoloxidase activity, hemocyte number, and encapsulation rate, were negatively affected by high density. Lindsey et al. (2009) [7] found that monarch butterflies in moderate- and high-density treatments also suffered an increase in infection. Goulson and Cory (1995) [8] also indicated that *M. brassicae* larvae reared at high density were more susceptible to disease than those reared at low density. In addition, Adamo (2006) [9] reported that crowding did not affect disease resistance in field crickets. Because insect immunity strongly affects the stability of host–parasite interactions, understanding any effects of larval density on a host’s immune response (or resistance) to natural enemies could help to elucidate its control effect and epidemic and population dynamics [10,11].

The diamondback moth (DBM), *Plutella xylostella* (Linn.), is one of the most destructive insect pests affecting cruciferous crops worldwide [12,13,14]. It has been reported that the total annual management costs combined with crop losses attributable to DBM have been estimated to be 4 to 5 billion US dollars [15]. It would be useful to develop integrated pest management programs for DBM, especially biological control of its natural enemies [16]. It has been reported that *Plxy* GV can easily develop epizootics in DBM populations and control their numbers effectively [12]. Host susceptibility to viruses plays an important role in viral infection and epizoosis [17]. However, any effects of population density on the immune response can influence host susceptibility. It has been found that in a field environment, parasitism by *Cotesia plutellae* at a lower larval density was higher than that at a higher larval density of *P. xylostella* [18].

Therefore, we hypothesized that *P. xylostella* larval populations at different densities exhibit different immune capacities for viruses. The aim of this study was to investigate the resistance to *Plxy* GV and the immune function of larvae reared at different densities under laboratory conditions. In this report, we describe the effects of larval density on (1) the survival of larvae infected by *Plxy* GV and (2) immunological variables of non-challenged and challenged larvae under laboratory conditions.

## 2. Materials and Methods

### 2.1. Insects

A laboratory colony of *P. xylostella* was initiated with eggs provided by Professor Youjun Zhang from the Institute of Vegetables and Flowers of the Chinese Academy of Agricultural Sciences. The hatched larvae were continuously reared on radish seedlings in the laboratory without exposure to any insecticide. Adults were provided with cotton balls soaked in 10% honey solution as food. The rearing conditions were 24 ± 2 °C and 70–80% relative humidity (RH) under a photoperiod of 14:10 h (light/dark).

### 2.2. Test Subjects

The *P. xylostella* colony was maintained for three generations, and the larvae were maintained at a regular density (100 larvae/box (30 × 20 × 10 cm)) on radish seedlings before experiments. Newly hatched larvae were grouped and reared at six density treatments of 1, 2, 5, 10, 15 and 20 larvae per Petri dish (diameter 6 cm). For all the density treatments, the number of larvae was maintained throughout the feeding period. Excess food was provided by adding fresh leaves every morning. All insects were maintained at a constant temperature of 24 ± 2 °C, 70–80% RH and photoperiod of L14:D10.

### 2.3. Mortality Exposure to Entomopathogenic Granulosis Virus

Granulosis virus of *P. xylostella* was obtained from Baiyun Industry Company (Jiyuan, Henan, China). The virus occlusion bodies (OBs) were purified by 20–60% sucrose gradient centrifugation by a protocol described by Meng and Ye (1996) [19]. Approximately 3.0 g of virus-infected larvae was used for purification. OBs were enumerated by a hemocytometer under 400X magnification. Prior to counting, OBs were diluted in ddH_2_O, and 5 µL of suspension was placed onto the center of a counting chamber. For the biossays, the final concentration was adjusted to 4.2 × 10^7^ OBs mL^−1^. Preliminary experiments demonstrated that this dose led to approximately 30% mortality.

When most larvae of a treatment density had developed to the third instar, the mortality exposure to granulosis virus was examined. The leaf discs (5 cm in diameter) were dipped in the granulosis virus concentration of 4.2 × 10^7^ OBsmL^−1^ for 10 s, air-dried and placed in Petri dishes with wet filter paper. Control leaf discs were treated with sterile water alone. Four replicates were established for per treatment. The larvae were allowed to feed on the treated disc for 24 h at 24 ± 2 °C and 70–80% RH under a photoperiod of 14:10 h (light: dark). After 24 h, the larvae were transferred to nontoxic leaf discs and reared until the larvae pupated or died under the same environmental conditions as described above. In addition, the survival rate was recorded every day.

### 2.4. Immune Assays

#### 2.4.1. Hemolymph Sampling

For the non-challenged larvae, hemolymph was sampled on the first day of the fourth instar, and larvae were cooled to torpor on ice and subsequently sterilized by swabbing with 75% ethanol. An abdominal proleg was cut with a fine scalpel, and hemolymph was collected by microcapillary pipette and transferred into a 1.5-mL centrifuge tube on ice to prevent melanization. For the challenged larvae, when most larvae of a treatment density had developed to the third instar, the larvae were treated by the same methods as described above in Section 2.3. Hemolymphs were collected on the second day after treatment. Both hemolymph samples collected from groups of 30 larvae per density were pooled as one replicate for their phenoloxidase activity (PO), lysozyme activity, antibacterial activity and total hemocyte counts to be determined.

#### 2.4.2. Phenoloxidase Activity

PO activity was tested as described by Wilson et al. (2001) [4]. Briefly, 50 µL of hemolymph was added to 40 µL of sodium phosphate solution in a plastic tube. Hemolymph PO activity was assayed spectrophotometrically by adding 50 µL of 0.02 M L-Dopa to 90 µL of hemolymph phosphate solution, and the mixture was incubated for 1 min at 22 °C. Absorbance was read at 490 nm on a temperature-controlled INFINITE200 microplate reader after 1 min of incubation. Protein content was measured in the samples following the method described by Bradford (1976) [20]. PO activity was expressed as PO units per milligram of protein, where 1 U was the amount of enzyme required to increase the absorbance by 0.001 min^−1^.

#### 2.4.3. Lysozyme Activity

Lysozyme activity was determined using a lysozyme kit (Nanjing Jiancheng Bioengineering Institute, Nanjing, China). First, 20 µL of hemolymph was mixed in a test tube with 2 mL of bacterial culture medium, and the two absorbance values at 530 nm were determined in a UV-2000 spectrophotometer (Unico Instrument Company of Shanghai, Shanghai, China) after 20 and 140 s. The lysozyme activity was determined using the two values according to the manufacturer’s instructions.

#### 2.4.4. Antibacterial Activity

Antibacterial activity against *Escherichia coli* was determined using an inhibition zone assay. Test plates were prepared according to Wilson et al. (2002) [21]. Next, 50-µL liquid cultures containing *E. coli* were added to Petri dishes filled with Luria broth medium. Holes (diameters 2 mm) were made by punching the agar layers and filling them with 15 µL of hemolymph. The plates were incubated at 37 °C for 24 h and then photographed. The diameters of clear zones were measured using IMAGE PRO PLUS 6.0. Because there was a linear correlation between the antibacterial activity of hemolymph and the diameter of the inhibition zones, the antibacterial activity of the hemolymph was determined by the diameter of the inhibition zones.

### 2.5. Total Hemocyte Counts

Total hemocyte counts were determined by adding 1 µL hemolymph and mixing with 9 µL phosphate buffered saline, and the hemocytes were counted in the central square and four corners of a hemocytometer under phase-contrast illumination and averaged to provide an estimate of the number of hemocytes.

### 2.6. Data Analyses

Data for comparison of two treatments were analyzed by Student’s paired *t*-test. Data for comparison of three or more groups were analyzed using one-way analysis of variance (ANOVA) with Tukey’s Honestly Significant Difference (HSD) for multiple testing. The survival was analyzed by Kaplan–Meier survival curves for the larvae in each group. The curves were compared using a log-rank test to determine which curves were significantly different from one another. All of the statistical analyses were undertaken using SPSS 19.0 [22].

## 3. Results

### 3.1. Survival after Challenge with Entomopathogenic Granulosis Virus

**Figure 1:**
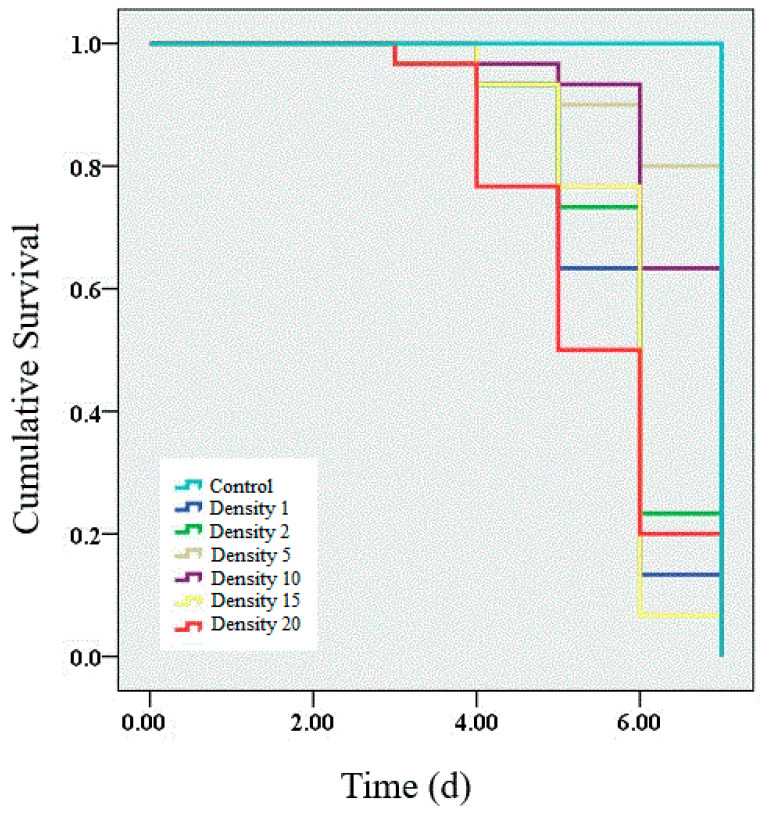
Survival analysis of larvae infected with granulosis virus at different larval densities. The average survival rates of larvae infected for 7 d at densities of 1, 2, 5, 10, 15, and 20 larvae per Petri dish were 63.33%, 66.67%, 70.00%, 66.67%, 73.33% and 76.67%, respectively. A log-rank test was used to determine which curves were significantly different from each other. Survival analysis was performed using the Kaplan–Meier estimator in SPSS 19.0.

We performed a survival analysis on the larvae infected by granulosis virus from densities of 1, 2, 5, 10, 15 and 20 larvae per Petri dish. The results showed that the survival rate of infected larvae was significantly decreased within 7 d of infection (Figure 1). Lower survival rates (63.33%, 66.67%, 70.00% and 66.67%) were observed in infected larvae at densities of 1, 2, 15 and 20 larvae per Petri dish than at densities of 5 (73.33%) and 10 (76.67%) larvae per Petri dish. Significant differences were observed among all the densities (χ^2^ = 47.56, *p* < 0.01). Unsurprisingly, no mortality was observed in the control group.

### 3.2. Enzyme Activity

#### 3.2.1. Phenoloxidase (PO) Activity

**Figure 2:**
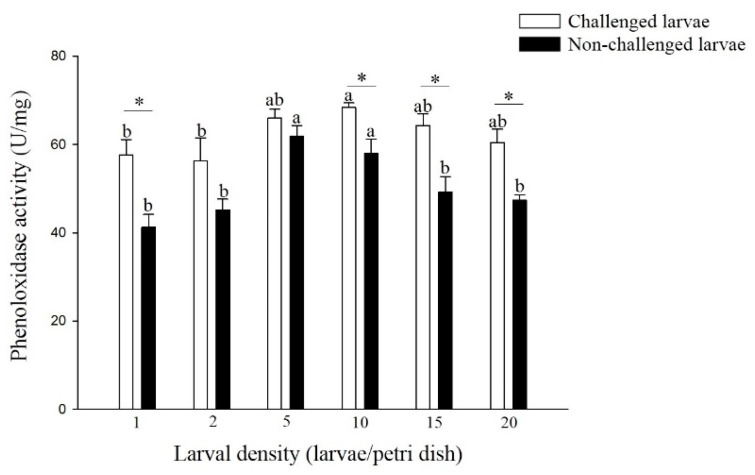
Phenoloxidase (PO) activity of larval *P. xylostella* reared at different larval densities. Bars of non-challenged (or challenged) larvae with the same letter are not significantly different at the 0.05 level by Tukey’s HSD multiple comparison. The asterisk indicates a significant difference at the 0.05 level by Student’s *t*-test between the non-challenged and challenged groups.

The hemolymph PO activity differed significantly among different densities of non-challenged and challenged larvae (Figure 2). The PO activity of non-challenged larvae reared at 5 and 10 larvae per Petri dish was significantly higher than that of larvae reared at the other density treatments (F = 8.27; df = 5, 17; *p* < 0.05). A similar trend was observed in larvae challenged with different densities (F = 2.36; df = 5, 17; *p* > 0.05), and the challenged larvae from the density of 10 larvae per Petri dish had significantly higher PO activity than did the larvae from the densities of 1 and 2 larvae per Petri dish. Among all the larval density treatments, the PO activity of the challenged larvae was higher than that of the non-challenged larvae, and there was a significant difference in the larval densities of 1, 10, 15, and 20 larvae per Petri dish (density 1: t = 3.59; df = 4; *p* < 0.05, density 10: t = 3.11; df = 4; *p* < 0.05, density 15: t = 3.46; df = 4; *p* < 0.05, density 20: t = 4.04; df = 4; *p* < 0.05).

#### 3.2.2. Lysozyme Activity

**Figure 3:**
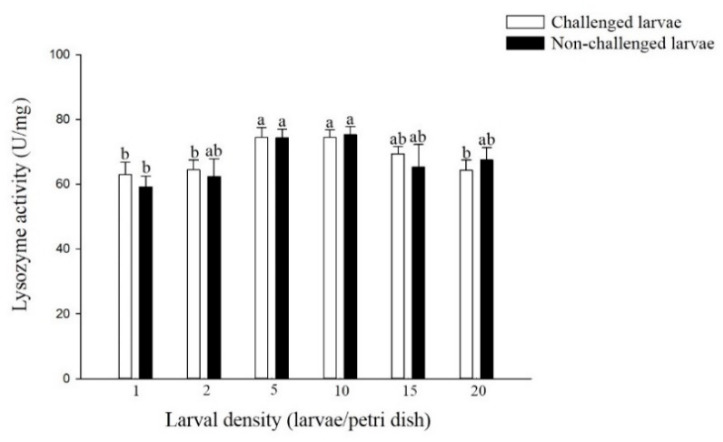
Lysozyme activity of larval *P. xylostella* reared at different larval densities. Bars of non-challenged (or challenged) larvae with the same letter are not significantly different at the 0.05 level by Tukey’s HSD multiple comparison.

The lysozyme activity challenged larvae reared at densities of 5 and 10 larvae per Petri dish was significantly higher than that of larvae reared at densities of 1, 2 and 20 larvae per Petri dish (F = 3.05; df = 5, 17; *p* < 0.05) (Figure 3). A similar trend was observed in non-challenged larvae with different densities (F = 2.09; df = 5, 17; *p* > 0.05), although no significant difference was observed in non-challenged larvae from most of the density treatments. In addition, there was no significant difference in the lysozyme activity of larvae from the six density treatments of challenged and non-challenged larvae.

#### 3.2.3. Antibacterial Activity

**Figure 4:**
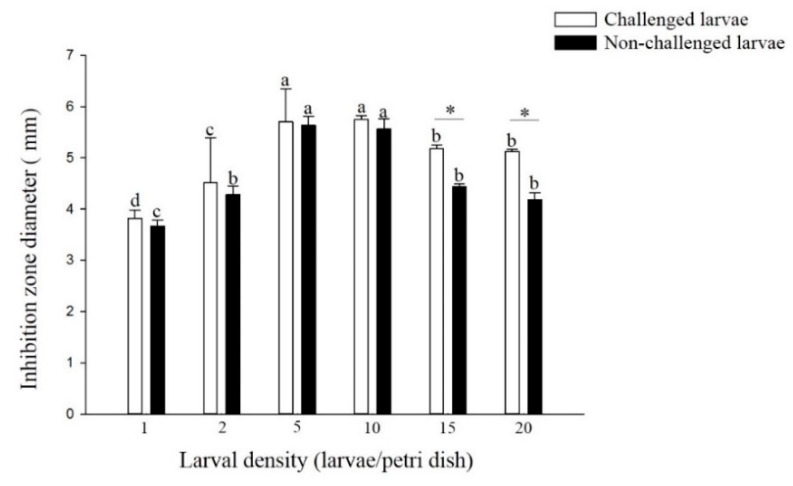
Antibacterial activity of larval *P. xylostella* reared at different larval densities. Bars of non-challenged (or challenged) larvae with the same letter are not significantly different at the 0.05 level by Tukey’s HSD multiple comparison. The asterisk indicates a significant difference at the 0.05 level by Student’s *t*-test between the non-challenged and challenged groups.

The antibacterial activity of the non-challenged and challenged larvae reared at densities of 5 and 10 larvae per Petri dish was significantly higher than that of larvae reared at other densities (non-challenged: F = 27.47; df = 5, 17; *p* < 0.01, challenged: F = 64.31; df = 5, 17; *p* < 0.01) (Figure 4). There was no significant difference in the antibacterial activity of larvae from the densities of 1, 2, 5 and 10 larvae per Petri dish treatment between the challenged and non-challenged larvae. However, among the higher densities of 15 and 20 larvae per Petri dish, the antibacterial activity of challenged larvae was significantly higher than that of non-challenged larvae (density 15: t = 7.88; df = 4; *p* < 0.01, density 20: t = 6.39; df = 4; *p* < 0.01).

### 3.3. Total Hemocyte Counts

**Figure 5:**
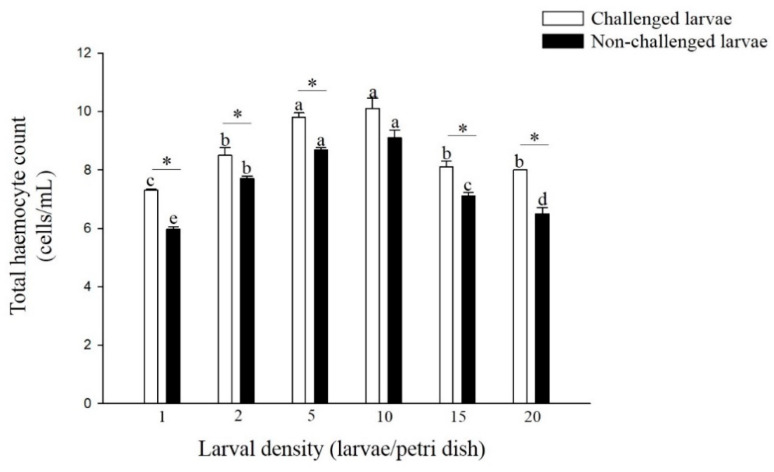
Total hemocyte counts of larvae *P. xylostella* reared at different larval densities. Bars of non-challenged (or challenged) larvae with the same letter are not significantly different at the 0.05 level by Tukey’s HSD multiple comparison. The asterisk indicates a significant difference at the 0.05 level by Student’s *t*-test between the non-challenged and challenged groups.

The total hemocyte counts (THC) of non-challenged and challenged larvae reared at 5 and 10 larvae per Petri dish was significantly higher than those reared at the other four densities (F = 59.16; df = 5, 17; *p* < 0.01, F = 25.53; df = 5, 17; *p* < 0.01) (Figure 5). The THC of challenged larvae was significantly higher than that of non-challenged larvae from all six density treatments, except for the density of 10 larvae per Petri dish (density 1: t = 13.15; df = 4; *p* < 0.01, density 2: t = 2.95; df = 4; *p* < 0.05, density 5: t = 6.33; df = 4; *p* < 0.01, density 15: t = 4.11; df = 4; *p* < 0.05, density 20: t = 5.81; df = 4; *p* < 0.01).

## 4. Discussion

Our present results showed that *P. xylostella* larvae exhibited density-dependent prophylaxis to their granulosis virus within a certain limited density. In the granulosis virus infection bioassay, the survival rate of *P. xylostella* larvae reared at densities of 5 and 10 larvae per Petri dish was significantly higher than that of larvae reared individually. This finding was consistent with previous reports on *S. littoralis*, *M. separata*, and *L. migratoria* [1,4,5], in which the survival rate of larvae challenged by entomopathogenic fungi and viruses was significantly increased in larvae reared at higher densities. In addition, in our previous study, we also found that the survival rate of non-challenged *P. xylostella* larvae (control) from higher larval density was higher than those larvae from lowest (or highest) density treatments [23]. Wilson and Reeson (1998) [3] predicted that DDP may extend to any invertebrate taxon that regularly experiences wide fluctuations in population density between generations. There were also population fluctuations in *P. xylostella* in different years or different seasons [24]. Thus, these results suggested that DDP is also exhibited in some insects characterized by wide population density fluctuations. Our findings provide evidence for DDP in non density-dependent phase-polyphenic insects. This study provides a new perspective for biocontrol effects and guide to improve the control strategy in different density populations of DBM.

Similar to pathogenic infection, a high population density alters insect prophylactic immunity. The immune system plays an important role in insect prophylactic resistance to various microorganisms [4]. The PO cascade is a suite of enzymes and plays crucial roles in the immune response, including the antiviral process [25]. Our results showed that the PO activity of larvae from 5 and 10 larvae per Petri dish was significantly higher than that of larvae from 1 and 2 larvae per Petri dish, regardless of whether the larvae were challenged. This finding was consistent with observations on *S. exempta* and *M. separata* in which the PO activity of larvae from higher density was significantly increased compared with those from solitary conditions [1,4]. In addition, compared with the hemolymph of the non-challenged larvae, the hemolymph of the challenged larvae had higher PO activity. This increased PO activity was also found in the hemolymph of *Helicoverpa zea* larvae inoculated with multiple nucleopolyhedroviruses and a single nucleopolyhedrovirus [26]. All these results suggested that PO activity plays an important role in larval resistance to granulosis virus at different densities.

Antimicrobial peptides, including lysozyme, were reported to play a critical role in the antibacterial and antiviral immunity of insects [27]. Our results indicated that the lysozyme activity of *P. xylostella* larvae challenged and non-challenged larvae from higher densities (5 and 10 larvae per Petri dish) was significantly higher than that of larvae from lower densities (1 and 2 larvae per Petri dish). Kong et al. (2016) [28] found that the expression level of the lysozyme gene of *L. sticticalis* larvae from higher density (10 larvae per jar) was significantly higher than that of larvae from lower density (1 larvae per jar). There was the same change trend in the antibacterial activity of challenged and non-challenged *P. xylostella* larvae from different density treatments. Wilson et al. (2002) [21] found that compared with solitary *L. migratoria*, gregarious *L. migratoria* had significantly higher antibacterial activity. Kong et al. (2019) [29] also reported that the antibacterial activity against Gram-positive *Staphylococcus aureus* and *Bacillus subtilis* and Gram-negative *Edwardsiella ictaluri* and *Vibrio anguillarum* in the hemolymph of *M. separata* larvae from high density (10 larvae per jar) was significantly higher than those from low density (1 larva per jar), and the relative expression of antimicrobial peptides (AMPs) gloverin, defensin, cecropin, lebocin and attacin related genes was significantly increased at higher density. Thus, the lysozyme activity and antibacterial activity of larvae may have an important effect on larval DDP. In addition, there was no significant difference in the lysozyme activity and antibacterial activity of larvae from most densities between challenged and non-challenged larvae. This finding may be related to the time after virus infection. In our study, lysozyme activity and antibacterial activity were tested after 48 h of virus infection, which was the early stage of virus infection. Lü et al. (2018) [30] found that there was no significant variation in the transcription level of the *C-lysozyme* gene of *Bombyx mori* cells during the nucleopolyhedrovirus early stage (48 h after infection).

The cellular response is mediated by hemocyte in the hemolymph [31]. Total hemocyte counts are important means of assessing the efficacy of the immune response [32,33]. Our results showed that regardless of whether the larvae were challenged or not challenged, the THC of larvae from higher densities (5 and 10 larvae per Petri dish) was significantly higher than that of larvae from lower densities (1 and 2 larvae per Petri dish). Although these findings do not agree with lone-reared caterpillars having an increased encapsulation response compared with group-reared caterpillars [34,35], they match the following findings. Yang et al. (2013) [36] found that the number of THC increased significantly as larval rearing density increased in *L. sticticalis* larvae. Kong et al. (2018) [1] also found that the THC of larvae from a higher density (10 larvae per jar) was significantly higher than those of larvae from a lower density (1, 2, 5 larvae per jar). In addition, the THC of challenged larvae was significantly higher than that of non-challenged larvae from most density treatments. Kiran Kumar and Singh (2011) [37] also found that THC increased in *Antheraea mylitta* larvae within 48 h after inoculation with cytoplasmic polyhedrosis virus. Thus, THC plays an important role in larvae from different density treatments against granulosis virus.

However, our results also suggested that the resistance and immune response of overcrowded larvae are not always higher than those of larvae from moderate-density conditions. In our study, the survival rate and immune parameters (PO activity, lysozyme activity, antibacterial activity and THC) of larvae decreased significantly when the density exceeded 15 larvae per Petri dish. Goulson and Cory (1995) [8] also found that the survival of *M. brassicae* larvae inoculated with nuclear polyhedrosis virus from the highest density of 20 larvae per pot was significantly lower than that from the higher density of 10 larvae per pot. Kong et al. (2013) [38] also found that the resistance to fungus *Beauveria bassiana* and parasitoid *Exorista civilis* of larvae from highest density was significantly lower than those of larvae from higher density. Kong et al. (2018) [1] also found that the THC and PO activity of *M. separata* larvae from highest density conditions were higher than those of larvae from high density conditions. All of these results indicated that the resistance of larvae from overcrowded conditions decreases. This decreased resistance to pathogens of larvae may be associated with larval competition for food, which could impact the immune response of overcrowded larvae even with food *ad libitum*. Kong et al. (2013) [38] found that the weight of pupae from crowded conditions is much less than that of pupae from other density conditions in *P. xylostella*. The trade-off between immune function and life history at high temperature also emerged in *Tenebrio molitor* [39].

Any effects of density on the immune response can influence population resistance to pathogens (or parasites). In this study, we found that compared with larvae from lower densities, larvae reared at moderate density showed significantly higher immune capacity to *Plxy* GV. It was found that in a field environment, the parasitism by *C. plutellae* at a higher larval density was lower than that at a lower larval density of *P. xylostella* [18]. This lower parasitism in higher density conditions may be correlated with high immune capacity to *C. plutellae*. Therefore, in the field, larval density may play an important role in the control effect of *P. xylostella* through its effects on immune function. This study provides a new perspective for understanding the biocontrol effect of different density populations of *P. xylostella* by granulosis virus and guiding improvements future management strategy.

## 5. Conclusions

Overall, our results showed that larval density had a significant effect on the resistance of *P. xylostella* larvae to entomopathogenic granulosis virus. Larvae reared at the medium density of 10 larvae per Petri dish had the highest survival rate and longest survival time. Both non-challenged and challenged larvae from this density also had significantly higher levels of PO activity, lysozyme activity, antibacterial activity and THC than those from other density treatments. These results provide basis for the larval resistance to granulosis virus in the laboratory. However, further studies should investigate the resistance of larvae from high or low density to pathogen resistance in the field.

## References

[1] Kong H.L., Dong C.L., Tian Z., Mao N., Wang C., Cheng Y.X., Zhang L., Jiang X.F., Luo L.Z. (2018). Altered immunity in crowded *Mythimna separata* is mediated by octopamine and dopamine. Sci. Rep..

[2] Anderson R.M., May R.M. (1981). The population dynamics of microparasites and their invertebrate hosts. Philos. Trans. R. Soc. B.

[3] Wilson K., Reeson A.F. (1998). Density-dependent prophylaxis: Evidence from Lepidoptera-baculovirus interactions?. Ecol. Entomol..

[4] Wilson K., Cotter S.C., Reeson A.F., Pell J.K. (2001). Melanism and disease resistance in insects. Ecol. Lett..

[5] Wang Y.D., Yang P.C., Cui F., Kang L. (2013). Altered immunity in crowded Locust reduced fungal (*Metarhizium anisopliae*) pathogenesis. PLoS Pathog..

[6] Piesk M., Karl I., Franke K., Fischer K. (2013). High larval density dose not induce a prophylactic immune response in a butterfly. Ecol. Entomol..

[7] Lindsey E., Metha M., Dhulipala V., Oberhauser K., Altizer S. (2009). Crowding and disease: Effects of host density on response to infection in a butterfy-parasite interation. Ecol. Entomol..

[8] Goulson D., Cory J.S. (1995). Responses of *Mamestra brassicae* (Lepidoptera, Noctuidae) to crowding-interactions with disease resistance, color phase and growth. Oecologia.

[9] Adamo S.A. (2006). The emergency life-history stage and immunity in the cricket, *Gryllus texensis*. Anim. Behav..

[10] Reilly J.R., Hajek A.E. (2008). Density-dependent resistance of the gypsy moth Lymantria dispar to its nudeopolyhedrovirus, and the consequences for population dynamics. Oecologia.

[11] Stavely F.J.L., Pell J.K., Chapman B., Glare T.R., Yeo H., Suckling D.M., Walter M. Insect pathogens for biological control of the diamondback moth with particular emphasis on the fuguns *Zoophthora radicans* in New Zealand. Proceedings of the 4th international Workshop.

[12] Talekar N.S., Shelton A.M. (1993). Biology, ecology, and management of the diamond back moth. Annu. Rev. Entomol..

[13] Sun D., Guo Z.J., Liu Y., Zhang Y.J. (2017). Progress and prospects of CRISPR/Cas systems in insects and other arthropods. Front. Physiol..

[14] Furlong M.J., Wright D.J., Dosdall L.M. (2013). Diamondback moth ecology and management: Problems, progress, and prospects. Annu. Rev. Entomol..

[15] Zalucki M.P., Furlong M.J., Srinivasan R., Shelton A.M., Collins H.L. (2011). Predicting outbreaks of a migratorypest: An analysis of DBM distribution and abundance revisited. Management of the Diamondback Moth and Other Crucifer Insect Pests: Proceedings of the Sixth International Workshop.

[16] Sarfraz M., Keddie A.B., Dosdall L.M. (2005). Biological control of the diamondback moth, *Plutella xylostella*: A review. Biocontrol. Sci. Technol..

[17] Guo Z.J., Kang S., Sun D., Gong L.J., Zhou J.L., Qin J.Y., Guo L., Zhu L.H., Bai Y., Ye F. (2020). MAPK- dependent hormonal signaling plasticity contributes to overcoming Bacillus thuringiensis toxin action in an insect host. Nat. Commun..

[18] Haseeb M., Kobori Y., Amano H., Nemoto H. (2001). Population density of Plutella xylostella (Lepidoptera: Plutellidae) and its parasitoid Cotesia plutellae (Hymenoptera: Braconidae) on two varieties of cabbage in an urban environment. Appl. Entomol. Zool..

[19] Meng X.L., Ye L.B. (1996). Research on synergistic factor of *Plutella xylostella* granulosis virus. J. Wuhan Univ..

[20] Bradford M.M. (1976). A rapid and sensitive method for the quantitation of microgram quantities of protein utilizing the principle of protein-dye binding. Anal. Biochem..

[21] Wilson K., Thomas M.B., Blanford S., Doggett M., Simpson S.J., Moore S.L. (2002). Coping with crowds: Density-dependent disease resistance in desert locusts. Proc. Natl. Acad. Sci. USA.

[22] Landau S., Everitt B.S. (2004). A Handbook of Statistical Analyses Using Spss.

[23] Kong H.L., Zhang Y.X., Zhu S.D., Kong Y., Wu L., Hu R.L. (2013). Effects of larval density on growth, development and reproduction of diamondback moth (DBM), *Plutella xylostella* (L.). Chin. J. Eco Agric..

[24] Feng X., Li Z.Y., Wu Q.J., Shen A.D., Wu Y.D., Hou Y.M., He Y.R., Li J.H., Xie S.H., Zhang J.M. (2011). Research progress of the resistance management and sustainable control of diamondback moth (*Plutella xylostella*) in China. J. Appl. Entomol..

[25] Nappi A.J., Vass E. (1993). Melanogenesis and the generation of cytotoxic molecules during insect cellular immune-reactions. Pigment Cell Res..

[26] Pan Q.J., Shikano I., Felton G.W., Liu T.X., Hoover K. (2020). Host permissiveness to baculovirus influences time-dependent immune responses and fitness costs. Insect Sci..

[27] Ren Q., Zhao X.F., Wang J.X. (2009). Molecular characterization and expression analysis of a chicken-type lysozyme gene from housefly (Musca domestica). J. Genet. Genom..

[28] Kong H.L., Lv M., Mao N., Wang C., Cheng Y.X., Zhang L., Jiang X.F., Luo L.Z. (2016). Molecular characterization of a lysozyme gene and its altered expression profile in crowded Beet Webworm (*Loxostege sticticalis*). PLoS ONE.

[29] Kong H.L., Dong C.L., Jing W.H., Zheng M.Y., Tian Z., Hou Q.L., Wang C., Cheng Y.X., Zhang L., Jiang X.F. (2019). Transcriptomic insight into antimicrobial peptide factors involved in the prophylactic immunity of crowded *Mythimna separata* larvae. Dev. Comp. Immunol..

[30] Lü P., Pan Y., Yang Y.H., Zhu F.F., Li C.J., Guo Z.J., Yao Q., Chen K.P. (2018). Discovery of anti-viral molecules and their vital functions in *Bombyx mori*. J. Invertebr. Pathol..

[31] Strand M.R. (2008). The insect cellular immune response. Insect Sci..

[32] De Andrade F.G., De Negreiro M.C.C., Levy S.M., De Batista Fonesca I.C., Moscardi F., Falleiros Â.M.F. (2010). Haemocyte quantitative changes in *Anticarsia gemmatalis* (Lepidoptera: Noctuidae) larvae infected by AgMNPV. Braz. Arch. Biol. Technol..

[33] Shaurub E.S.H., El-Meguid A.A., Abd El-Aziz N.M. (2014). Quantitative and ultrastructural changes in the haemocytes of *Spodoptera littoralis* (Boisd.) treated individually or in combination with *Spodoptera littoralis* multicapsid nucleopolyhedrovirus (SpliMNPV) and azadirachtin. Micron.

[34] Silva F.W.S., Viol D.L., Faria S.V., Lima E., Valicente F.H., Elliot S.L. (2013). Two’s a crowd: Phenotypic adjustments and prophylaxis in *Anticarsia gemmatalis* larvae are triggered by the presence of conspecifics. PLoS ONE.

[35] Silva F.W.S., Elliot S.L. (2016). Temperature and population density: Interactional effects of environmental factors on phenotypic plasticity, immune defenses, and disease resistance in an insect pest. Ecol. Evol..

[36] Yang Z.L., Cheng Y.X., Luo L.Z., Kong H.L., Zhang L., Lei C.L. (2013). Effects of larval density on the number and composition of hemocytes in the beet webworm, *Loxostege sticticalis* (Lepidoptera: Pyralidae). Acta Entomol. Sin..

[37] Kiran Kumar K.P., Singh G.P. (2015). Hemocyte and biochemical changes of *Antheraea mylitta* D. infected with *Antheraea mylitta* cytoplasmic polyhedrosis virus (*AmCPV*). IJSR.

[38] Kong H.L., Cheng Y.X., Luo L.Z., Sappington T.W., Jiang X.F., Zhang L. (2013). Density-dependent prophylaxis in crowded Beet Webworm, *Loxostege sticticalis* (Lepidoptera: Pyralidae) larvae to a parasitoid and a fungal pathogen. Int. J. Pest Manag..

[39] Prokkola J., Roff D., Karkkainen T., Krams I., Rantala M.J. (2013). Genetic and phenotypic relationships between immune defense, melanism and life-history traits at different temperatures and sexes in *Tenebrio molitor*. Heredity.

